# Spontaneous Crystallization of Perovskite Nanocrystals in Nonpolar Organic Solvents: A Versatile Approach for their Shape‐Controlled Synthesis

**DOI:** 10.1002/anie.201906862

**Published:** 2019-09-26

**Authors:** He Huang, Yanxiu Li, Yu Tong, En‐Ping Yao, Maximilian W. Feil, Alexander F. Richter, Markus Döblinger, Andrey L. Rogach, Jochen Feldmann, Lakshminarayana Polavarapu

**Affiliations:** ^1^ Chair for Photonics and Optoelectronics, Nano-Institute Munich Department of Physics Ludwig-Maximilians-Universität München (LMU) Königinstrasse 10 80539 Munich Germany; ^2^ Nanosystems Initiative Munich (NIM) and Center for NanoScience (CeNS) Schellingstrasse 4 80799 Munich Germany; ^3^ Department of Materials Science and Engineering, and Centre for Functional Photonics (CFP) City University of Hong Kong 83 Tat Chee Avenue, Kowloon Hong Kong S.A.R. China; ^4^ Department of Chemistry Ludwig-Maximilians-Universität München Butenandtstrasse 5–13 (E) 81377 Munich Germany

**Keywords:** FAPbX_3_ nanocubes, FAPbX_3_ nanoplatelets, ligand-assisted reprecipitation, perovskite nanocrystals, spontaneous crystallization

## Abstract

The growing demand for perovskite nanocrystals (NCs) for various applications has stimulated the development of facile synthetic methods. Perovskite NCs have often been synthesized by either ligand‐assisted reprecipitation (LARP) at room temperature or by hot‐injection at high temperatures and inert atmosphere. However, the use of polar solvents in LARP affects their stability. Herein, we report on the spontaneous crystallization of perovskite NCs in nonpolar organic media at ambient conditions by simple mixing of precursor–ligand complexes without application of any external stimuli. The shape of the NCs can be controlled from nanocubes to nanoplatelets by varying the ratio of monovalent (e.g. formamidinium^+^ (FA^+^) and Cs^+^) to divalent (Pb^2+^) cation–ligand complexes. The precursor–ligand complexes are stable for months, and thus perovskite NCs can be readily prepared prior to use. Moreover, we show that this versatile synthetic process is scalable and generally applicable for perovskite NCs of different compositions.

## Graphical abstract

**Faster than fast**: Shape‐controlled lead halide perovskite nanocrystals crystallize spontaneously at ambient conditions when precursor–ligand complexes are mixed directly in a nonpolar organic medium. Nanocubes and nanoplatelets have been obtained. This versatile synthetic process is generally applicable for a wide range of halide perovskite nanocrystals.

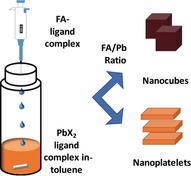

Over the last few years, halide perovskite nanocrystals (NCs) have emerged as a new class of semiconductor materials and efficient color‐tunable light sources for a wide range of applications such as displays, light‐emitting devices, lasers, broadband photodetectors, phototransistors, and photovoltaics owing to their extraordinary optical and optoelectronic properties, which are easily tunable by their composition and morphology.^1^ In addition, they offer several important features including ease of synthesis, tunable surface chemistry, near‐unity photoluminescence quantum yields (PLQY), access to quantum confinement effects, and solution processability, which are crucial for most device applications.^2^ Therefore, there has been increased interest in the facile synthesis of shape‐controlled perovskite NCs not only for a fundamental understanding of their structure–property relationships, but also to meet the demand for various technological applications.^3^

Since the first colloidal synthesis of perovskite NCs was reported in 2014, great efforts have been devoted to the fabrication of high‐quality perovskite NCs (hybrid organic–inorganic as well as all‐inorganic) by various synthetic methods such as ligand‐assisted reprecipitation (LARP),^4^ hot injection,1b, 5 ultrasonication,^6^ solvothermal,^7^ microwave,^8^ and ball‐milling.^9^ Among these methods, the LARP approach has received special attention because of its ability to produce perovskite NCs at room temperature.4a, 10 This synthesis method is based on the crystallization of perovskite precursors to form NCs in the presence of ligands in a good solvent by the addition of a bad solvent or vice versa. The bad solvent triggers the aggregation of perovskite precursors to form NCs, while the ligands dictate their dimensions. LARP has been widely applied to prepare perovskite NCs of different morphologies such as dots, platelets, and wires.1c, 3d, 4b However, the drawback of this method is that the use of polar solvents, such as *N*,*N*‐dimethylformide (DMF), greatly affects the stability of NCs.^11^ Moreover, this method may result in mixed NC morphologies, such as nanoplatelet dispersions of various thicknesses, leading to broad PL spectra. In contrast, perovskite NCs can be directly synthesized in nonpolar organic media, however, at relatively high temperature. Herein, we report an unconventional ligand‐assisted reprecipitation method that does not require external stimuli such as heat, microwave irradiation, ultrasonication, mechanical force, or polar solvent, in which perovskite NCs are easily obtained through spontaneous crystallization upon simple mixing of precursor‐ligand complexes in organic media at ambient atmosphere. The shape of the NCs can be tuned from nanocubes to nanoplatelets by varying the ratio of monovalent (e.g. formamidinium (FA^+^) and Cs^+^) to divalent cation (Pb^2+^) precursors in the reaction medium. Mechanistic studies reveal that the NCs are formed through seed‐mediated growth. Furthermore, we show that this simple synthetic approach is versatile.

As shown in Figure 1 a, our synthesis is based on simple mixing of two precursor complexes (monovalent cation (A^+^)–ligand and lead halide (PbBr_2_)–ligand) in a nonpolar solvent under ambient conditions, and at room temperature (see Movie S1 for visualization of shape‐controlled synthesis at room temperature). This approach is different from the conventional LARP method. Here, the precursors are directly dissolved in nonpolar solvents through metal ion complexation of ligands, and the crystallization occurs spontaneously when the two precursors are mixed. Importantly, we found that the precursor solutions are extremely stable for months and the perovskite NCs can be readily prepared in large quantities a few minutes prior to use. In principle, this facile synthetic approach could be easily applied to a range of ABX_3_ perovskite NCs (where A is a monovalent cation, B is lead, and X is a halide ion) with different types of A and X compositions as depicted in Figure 1 b. We first applied this approach to the synthesis of formamidium lead iodide perovskite NCs. In a typical synthesis, pre‐prepared FA–oleate precursor was injected into a toluene solution containing the PbI_2_–ligand complex under continuous stirring at room temperature (see the Supporting Information for details). The color of the reaction medium changes to orange and the reaction medium starts to fluoresce under UV light immediately after the two precursors are mixed without application of heat or use of polar solvent. This suggests that the precursor–ligand complexes break down and spontaneously crystallize to form FAPbI_3_ colloidal perovskite NCs in the reaction medium. In addition, the orange emission of FAPbI_3_ NCs indicates that charge carriers are quantum confined, since the bulk FAPbI_3_ exhibits near‐infrared emission (≈800 nm).^12^ Increasing the amount of FA–oleate (200 μL of 0.05 mol L^−1^) added to the PbI_2_–ligand solution yields a dark‐brown solution with red luminescence under UV light illumination, which indicates the formation of FAPbI_3_ colloidal NCs with weaker quantum confinement.

**Figure 1:**
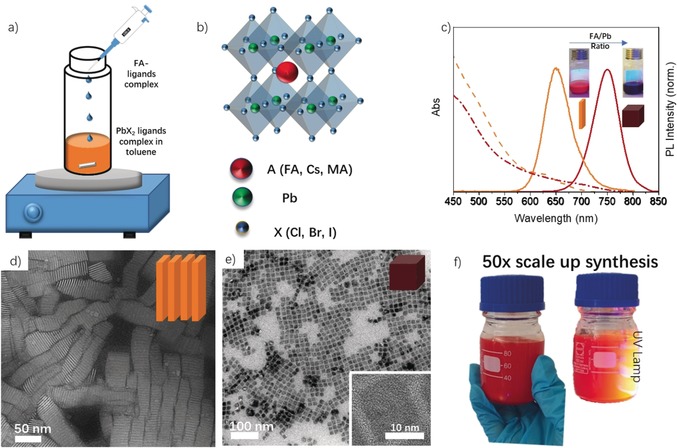
a) Schematic illustration of the synthesis of perovskite NCs by addition of a monovalent cation–ligand (e.g. FA–oleate) complex into the PbX_2_–ligand solution at ambient conditions. The reaction yields different types and compositions of perovskite NCs depending on the type of A and X. b) Schematic representation of the cubic crystal structure of ABX_3_ perovskite NCs. c) UV/Vis absorption (dashed lines) and PL spectra (solid lines) of perovskite NCs obtained by the addition of different amounts of FA–oleate precursor into the PbI_2_–ligand solution. The insets show photographs of the colloidal solutions under UV illumination. d,e) HAAD‐STEM image of nanoplatelets and TEM images of FAPbI_3_ perovskite nanocubes, respectively; the inset in (e) shows a high‐resolution TEM image of a single nanocube. f) Scaled‐up synthesis of perovskite NCs. Photograph of a FAPbI_3_ colloidal nanoplatelet dispersion (scaled up by a factor of 50) under room light (left) and UV light (right, *λ*=365 nm).

The optical properties of the obtained FAPbI_3_ colloidal solutions were characterized by UV/Vis absorption and PL spectroscopy (Figure 1 c). The colloidal dispersion exhibits an absorption onset at 625 nm and a single PL emission peak at 650 nm for NCs obtained with the addition of 50 μL FA–oleate, with a small Stokes shift (25 nm) and a full width at half maximum (FWHM) of 60–65 nm. The absorption edge and the emission peak are clearly redshifted to 720 nm and 750 nm, respectively, when an increased amount of FA–oleate (200 μL) was mixed with the PbI_2_–ligand solution. Therefore, the PL peak position of the NCs prepared by this simple approach is easily tunable by varying the ratio of FA to Pb precursor (Figure S1). This is in a way similar to the synthesis of CsPbBr_3_ NCs which show tunable emission when the ratio of Cs to Pb precursor is varied.3e Additionally, the PL spectra of strongly confined perovskite NCs (PL at 650 nm) exhibit a small shoulder at the red‐side of the spectrum, suggesting a minor polydispersity of the colloidal dispersion. This has already been observed in perovskite NCs prepared by other methods.

The morphology of the FAPbI_3_ NCs has been characterized by transmission electron microscopy (TEM). The corresponding images of the NCs obtained with the addition of 50 μL and 200 μL FA–oleate show nearly monodisperse nanoplatelets and nanocubes (Figure 1 d,e), respectively. This is in agreement with our previous study showing the transformation of morphology from 3D nanocubes to 2D nanoplatelets when the ratio of monovalent to bivalent precursor was decreased in an ultrasonication‐assisted synthesis of perovskite NCs.6a As shown in Figure 1 d,e, the nanoplatelets and nanocubes are highly monodisperse and tend to self‐assemble on the TEM grid with face‐face stacking and cubic close packing, respectively. The average thickness of the nanoplatelets determined from TEM is ≈2.3 nm, which corresponds to four octahedral monolayers; this suggested the PL peak wavelength of 650 nm was expected for nanoplatelets with a thickness of four monolayers. It is worth mentioning that the nanoplatelets obtained here exhibit higher quality and monodispersity compared to the nanoplatelets synthesized by the LARP method (Figure 1 d, see also Figure S2 for large‐area STEM images).^13^ The average edge length of the FAPbI_3_ nanocubes is ≈12–14 nm (size distribution is shown in Figure S3 inset) with a PL peak at 751 nm. The high‐resolution TEM image shows that the nanocubes are single‐crystalline (inset of Figure 1 e, large‐area STEM images are shown in Figure S3). The XRD spectra of FAPbI_3_ nanoplatelets and nanocubes show that both exhibit a cubic perovskite crystal structure (Figure S4). The nanoplatelets have broader XRD peaks compared to the nanocubes indicating a smaller grain size. The PLQYs of FAPbI_3_ nanoplatelets and nanocubes were found to be 30 % and 65 %, respectively. The lower PLQY of the nanoplatelets is because those are more susceptible to surface defects compared to nanocubes as they have larger surface‐to‐volume ratio. Furthermore, we demonstrate the scalability of this simple synthesis method by increasing each reaction component's volume by 50 times (Figure 1 f), producing 100 mL of FAPbI_3_ colloidal nanoplatelet dispersion in one run. The absorption, PL spectra, and PLQY are nearly same for the particles obtained from the larger scale synthesis (Figure S5). Our results clearly suggest that simple mixing of two precursors in different ratios results in shape control with excellent monodispersity and scalability. Additionally, we found that the NCs prepared by this approach exhibit higher long‐term stability than the NCs prepared by conventional LARP synthesis under UV illumination (*λ*=365 nm, 12 W power) at ambient conditions (see Figure S6).

In general, the growth of most metal and semiconductor NCs is seed‐mediated or stimulated by a template, while the shape of the NCs can be controlled through precursor concentrations, ligands, and reaction temperature. In order to elucidate the growth of FAPbI_3_ perovskite NCs upon mixing the two precursors, we performed the reaction in a cuvette and monitored the PL evolution of nanocrystals formed in the reaction medium over a period of time. As depicted in Figure 2 a, two sharp PL peaks appear at ≈620 nm and ≈640 nm immediately after the precursors are mixed, suggesting that the reaction is extremely fast and that the quantum‐confined perovskite NCs are already formed at the early stages of the reaction. These two PL peaks correspond to nanoplatelets with a thickness of three and four octahedral monolayers, respectively.3e, 6a After 2 s, the PL peak at 640 nm redshifts to ≈660 nm, while the peak at 620 nm remains unchanged. In addition, a new peak at 680 nm emerges and redshifts gradually to 730 nm over the next 5 s corresponding to the final product of nanocubes. At this stage, all other peaks at shorter wavelengths corresponding to strongly quantum‐confined NCs have vanished, meaning that the smaller particles grow faster than the larger ones, and thus eventually the reaction yields NCs of only one size, as evidenced from TEM images (Figure 1 e). It is difficult to characterize the morphology of intermediate particles due to the extremely fast reaction. However, the PL evolution of the nanocubes suggests a bimodal size distribution of clusters formed at early stages of the reaction, which then disappear as time progresses owing to their transformation into nanocubes through size focusing, as schematically shown in Figure 2 b.

**Figure 2:**
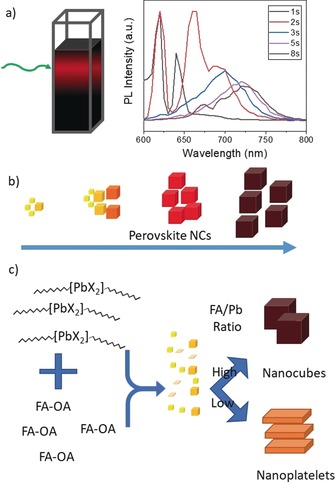
a) Probing the crystallization of perovskite nanocubes in nonpolar organic media by in situ PL measurements. b) Schematic illustration of the possible size distribution of perovskite NCs at different reaction times. c) Schematic illustration showing the transformation of precursor–ligand complexes into either FAPbX_3_ nanocubes or nanoplatelets depending on the FA/Pb ratio.

Although the PL evolution of the product gives insight into the possible growth mechanism, it is unclear how the precursors crystallize into perovskite nuclei when they are mixed in a nonpolar organic solution. To gain a better understanding of this process, one has to consider the dissolution of precursor salts in organic media. Generally, precursor metal ions are not soluble in organic solvents. However they can be solubilized using coordinating ligands through metal–ligand complexation, meaning that the ligand solution acts like a good solvent for the precursors. Here, perovskite complexes are made by dissolving corresponding salts in a ligand solution, which is then injected into toluene, a bad solvent for metal ion precursors. Therefore, the precursors crystallize into perovskite nuclei immediately when they are mixed in an organic solvent, like in the conventional LARP method (Figure 2 b). Further evidence comes from the fact that the crystallization does not occur when both precursor–ligand complexes are mixed in the ligand (oleylamine/oleic acid) solution due to the fact that ligand solution acts as coordinating solvent. It is likely that the energy difference between precursor–ligand complexes and perovskite NCs coated with a dense ligand shell is so small that the crystallization takes place spontaneously at room temperature immediately when they are mixed in organic media. As schematically depicted in Figure 2 c, the morphology of NCs changes from 3D nanocubes to 2D nanoplatelets when the amount of the FA precursor added to the reaction medium is decreased. This suggests that anisotropic growth is favorable at a lower ratio of monovalent to divalent cation (for instance, FA/Pb ratio). In general, decreasing the concentration of reactants decreases the rate of reaction, and thus nanocrystal growth rate. Moreover, a slow growth rate favors the formation of anisotropic NCs, which has been also observed previously for metal nanoparticles.^14^

We further verified the versatility of this facile synthesis approach by applying it to different kinds of perovskite NCs, such as CsPbI_3_ and CsPbBr_3_. This has been achieved by selecting the respective monovalent cation–oleate (e.g. Cs–oleate, which was prepared using cesium acetate) and PbX_2_–ligand complexes as reactants. As shown in Figure 3, CsPbBr_3_ and CsPbI_3_ nanoplatelets were successfully prepared by simply mixing Cs–oleate with a PbX_2_–ligand complex (X=Br or I) in toluene. The TEM images (Figure 3 a,b) show that these nanoplatelets are nearly monodisperse with average thicknesses of 1.1 nm and 1.2 nm, respectively, and they tend to self‐assemble on the TEM grid in a face–face stacking fashion. The absorption spectra show well‐resolved excitonic peaks with the PL centered at ≈475 nm and 620 nm for CsPbBr_3_ and CsPbI_3_ nanoplatelets, respectively (Figure 3 c,d). These PL peak positions correspond to four octahedral monolayers for both materials. Interestingly, a slightly higher reaction temperature (≈80 °C) was necessary to prepare CsPbX_3_ nanocubes when the two precursors were mixed in toluene (Figure S7), in contrast to the room‐temperature formation of FAPbI_3_ NCs. This is likely due to a higher energy barrier between perovskite precursors and CsPbBr_3_ nanocubes. Nevertheless, this reaction temperature is still far lower than the temperature (typical 180 °C) of the hot‐injection synthesis used to produce CsPbBr_3_ nanocubes in organic solvents.1b Furthermore, we have shown that this versatile approach can be applicable to the preparation of FAPbCl_3_, FAPbBr_3_, and MAPbBr_3_ NCs (Figure S8).

**Figure 3:**
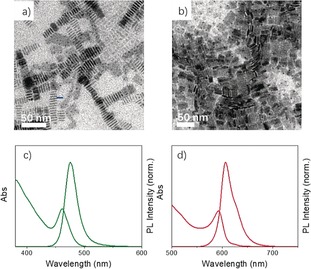
TEM images of a) CsPbBr_3_ and b) CsPbI_3_ nanoplatelets with their absorption and PL spectra shown in (c) and (d), respectively.

In conclusion, we have presented a facile scalable synthesis of perovskite NCs at ambient conditions by spontaneous crystallization in a nonpolar organic solvent. Compared to the classical LARP method, no polar solvent is needed to dissolve the precursors; instead, ligands act as coordinating solvents. The morphology of perovskite NCs is easily tunable from 3D nanocubes to 2D nanoplatelets by decreasing the ratio between the monovalent cation and Pb^2+^ precursors. Importantly, we have demonstrated the versatility of this synthesis approach by applying it to both organic–inorganic hybrid and all‐inorganic perovskite NCs of different halide compositions. We foresee that this facile method could be easily extended to obtain other morphologies such as nanorods or nanowires by varying the concentration and temperature of the Pb^2+^ precursor. This versatile and facile synthesis method not only opens new avenues toward the shape‐controlled perovskite NCs with excellent scalability, but also expands our current understanding of the crystallization of perovskite NCs directly in nonpolar solvents.

## Conflict of interest

The authors declare no conflict of interest.

## Supporting information

As a service to our authors and readers, this journal provides supporting information supplied by the authors. Such materials are peer reviewed and may be re‐organized for online delivery, but are not copy‐edited or typeset. Technical support issues arising from supporting information (other than missing files) should be addressed to the authors.

Supplementary

Supplementary
