# Supplementary material for: Spontaneous Crystallization of Perovskite Nanocrystals in Nonpolar Organic Solvents: A Versatile Approach for their Shape‐Controlled Synthesis
Source: Angew Chem Int Ed Engl. 2019 Sep 26;58(46):16558–62. doi: 10.1002/anie.201906862 (PMC6899919; doi:10.1002/anie.201906862)
Supplement: Supplementary file 1 — Supplementary [file ANIE-58-16558-s001.pdf]

Angewandte Chemie
Eine Zeitschrift der Gesellschaft Deutscher Chemiker

Supporting Information

Spontaneous Crystallization of Perovskite Nanocrystals in Nonpolar Organic Solvents: A Versatile Approach for their Shape-Controlled Synthesis

He Huang, Yanxiu Li, Yu Tong, En-Ping Yao, Maximilian W. Feil, Alexander F. Richter, Markus Döblinger, Andrey L. Rogach, Jochen Feldmann, and Lakshminarayana Polavarapu**

anie_201906862_sm_miscellaneous_information.pdf
anie_201906862_sm_Movie_S1.mp4

Supporting Information

Supporting Information

Materials: Formamidine acetate(99%), cesium acetate(99.9%), cesium carbonate (Cs_2CO_3, 99 %), methylammonium bromide (MABr, ≥99%), lead(II) iodide (PbI_2, 99 %), lead(II) bromide ($PbBr_2$, ≥ 98 %), oleic acid (technical grade 90 %), oleylamine (technical grade 70 %,), toluene (for HPLC, ≥ 99.9 %) were purchased from Sigma-Aldrich. All chemicals were used as received.

Preparation of precursors*:* formamidine-oleate (FA-oleate) precursor was prepared by dissolving 0.5 mmol formamidine acetate in 10 mL of oleic acid (concentration: 0.05 mol/L) under ultrasonication. The mixture was sonicated until all the precursor get dissolved. Cesium-oleate (Cs-oleate) precursor was prepared under similar experimental conditions using either Cesium carbonate or cesium acetate. PbX_2 (X=Br or I) precursor solution was prepared by dissolving 0.1 mmol PbX_2 powder in a mixture of 100 µl of oleic acid, 100 µl oleylamine and 10 mL toluene (concentration: ~0.01 mol/L) at 80 ^oC under continuous stirring.

Synthesis of $FAPbX_3$ nanocrystals: In a typical synthesis, 50 or 200 µl (for nanoplatelets or nanocubes, respectively) of formamidine-oleate was added into 2 mL of PbI_2 precursor (prepared in toluene) solution under vigorous stirring at room temperature. After 2 mins of stirring, the solution was centrifuged at 14500 rpm for 5 mins and the precipitate was redispersed in 1 mL of toluene. For monitoring the time dependent evolution of PL, the reaction was performed in a cuvette. Immediately after the addition of FA-oleate, the PL spectra of the reaction medium was monitored over a period of time. To probe the growth mechanism, the synthesis of $FAPbI_3$ NCs was carried out in a quette with a small magnetic stir bar in it, and it was placed in a PL spectrometer. In a typical reaction, 2 mL of PbI_2 precursor solution was added into a cuvette, followed by addition of formamidine-oleate. The PL spectrometer was set to acquire the PL spectra of reaction medium for every 1 s after the addition of formamidine-oleate in a fast scan mode. The acquired spectral data was automaticly saved by software.

Synthesis of $CsPbX_3$ nanocrystals: The process is similar to the synthesis of $FAPbX_3$, as mentioned above. While the nanoplatelets were prepared at room temperature, the reaction was performed at 80 ^oC to obtain $CsPbX_3$ nanocubes.

Synthesis of $MAPbBr_3$ nanocrystals: The synthetic process is similar to the synthesis of $FAPbX_3$, as mentioned above, but replacing FA-olate with MA-oleate. The MA-oleate was prepared by addition of MABr in oleic acid with concentration of 0.01 mol/L. The synthesis of $MAPbBr_3$ NCs was carried out by the addition of 100 µL of MA-OA to 2 mL of $PbBr_2$ precursor solution.

Characterization: The absorption and photoluminescence spectra were obtained with a Cary 60 UV-Vis spectrophotometer and a Varian Cary Eclipse fluorescence spectrophotometer (Agilent Technologies), respectively. The photoluminescence quantum yields (PLQYs) were determined by an absolute method using an integrating sphere with its inner face coated with BENFLEC® (Edinburgh Instruments) coupled to Fluorolog spectrofluorometer. The morphology of the NCs was characterized by transmission electron microscopy (TEM) operating at an accelerating voltage of 80-100 kV (JEOL JEM-1011). HAADF-STEM images were acquired using a cubed FEI Titan microscope operating at 300 kV. A probe semiconvergence angle of ~21 mrad was used. X-ray diffraction (XRD) measurements were performed by a Philips X-Pert Xray diffractometer using Cu Kα radiation (λ = 1.5418 Å).

Stability measurement: Herein we compared the changes of the normalized PL intensity of FAPbI3 by this method and LARP method. The synthesis of $FAPbI_3$ NCs by LARP method (using FABr and $PbBr_2$ as precursors) was carried by following the previous report by Zhang et al. (ACS Nano 2015, 9, 4533-4542). The changes of the normalized PL intensity of these different NCs UV-illuminated for up to 2 h are shown in Figure **S7** and are used as a measure of their stability. For $FAPbI_3$ NCs synthesized by LAPR method, the PL intensity drops fast within the 40 min. The reason for such a diminished stability of this kind of samples lies first in the chemical nature of the organic–inorganic perovskites, which are extremely sensitive to humidity and oxygen; this has been well documented in numerous stability studies conducted on both, thin films, and NCs. On top of that, it is believed that the presence of polar solvent DMF originating from the bottom-up chemical synthesis, with its much higher boiling point (153 °C) than that of toluene (110 °C), results in the dissolution/decomposition of the formed perovskite NCs back to precursors. Surprisingly, $FAPbI_3$ NCs prepared by this method as introduced in this paper showed superior stability. This very favorable performance is attributed to the absence of any destructive polar solvent, such as DMF in the samples fabricated in this work.

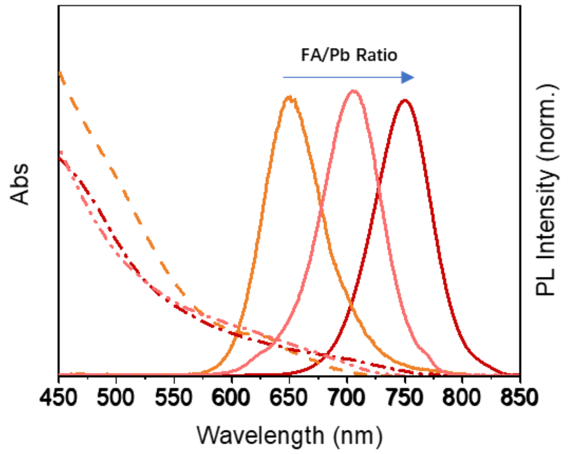

***Figure S1*:** Absorption and PL spectra of $FAPbI_3$ nanoplatelets with different amount of FA-oleate precursor into the PbI_2-ligand solution.

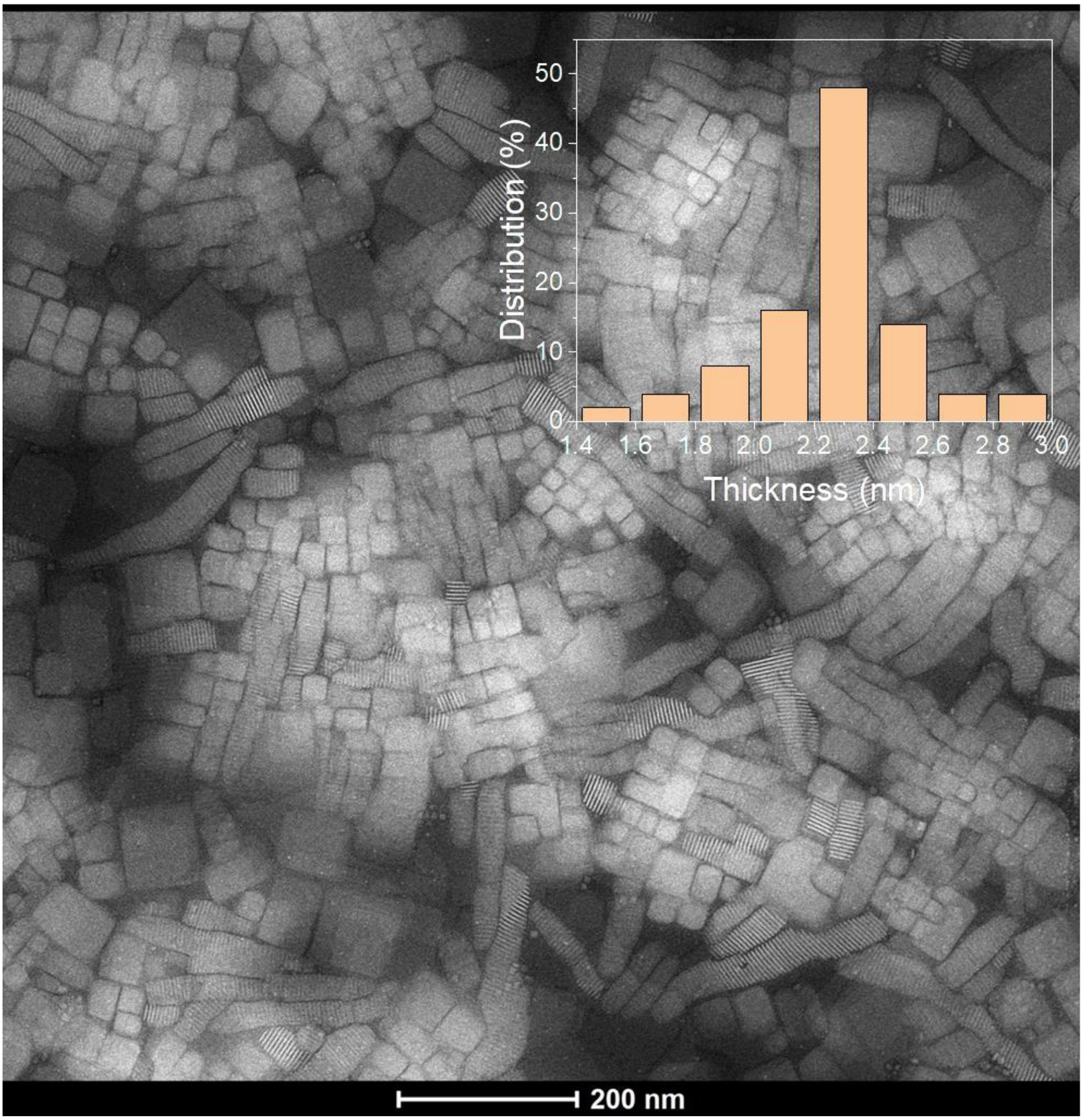

***Figure S2*:** Large area STEM image of $FAPbI_3$ nanoplatelets (Inset: Histogram of nanoplatelet thickness distribution)

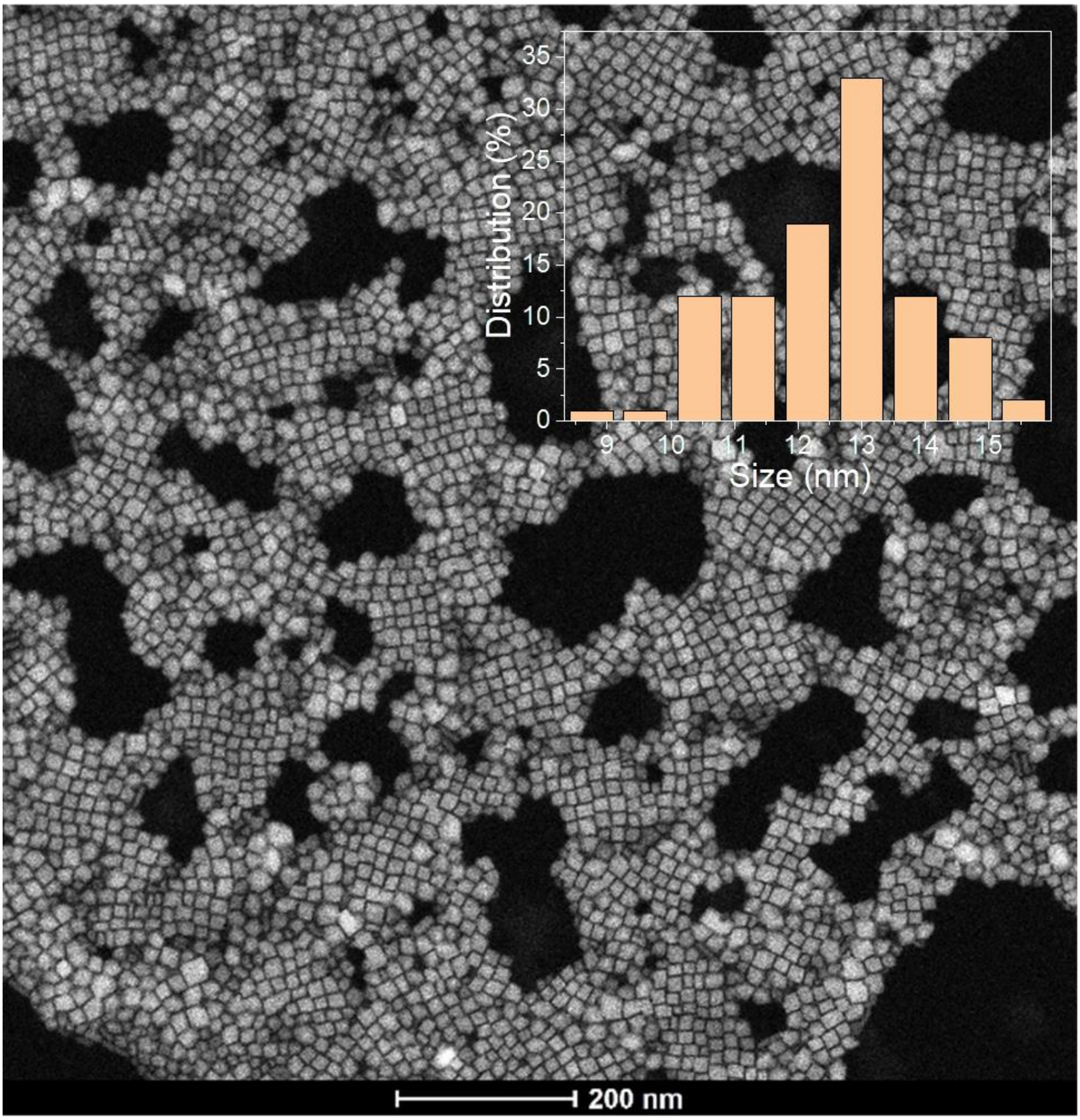

***Figure S3*:** Large area STEM image of $FAPbI_3$ nanocubes (Inset: Histogram of particle size distribution)

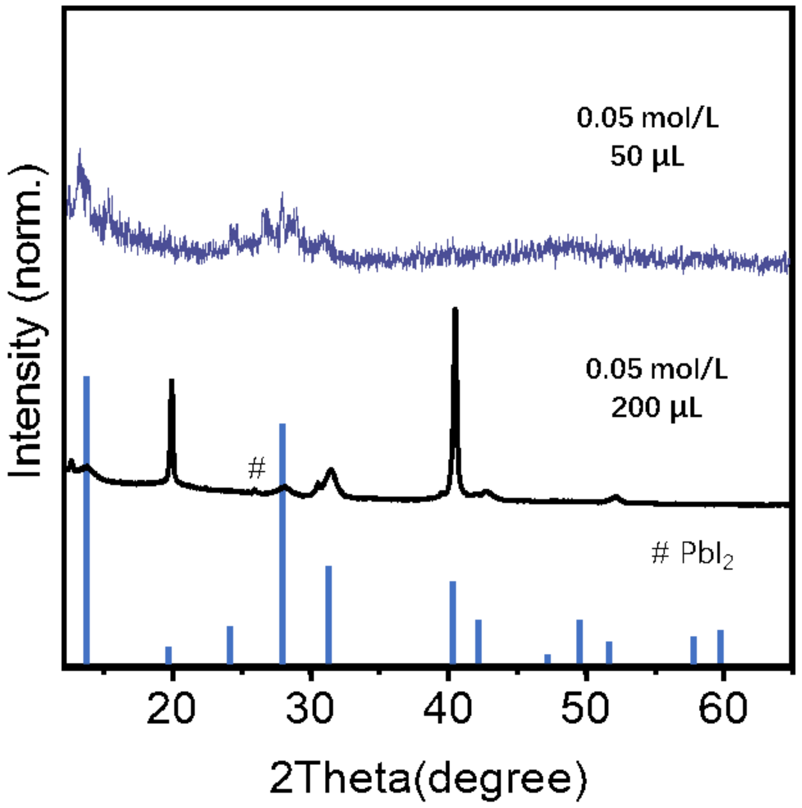

Figure S4: XRD data of $FAPbI_3$ perovskite nanocrystals prepared with different amounts of FA-oleate, along with the simulated pattern of bulk $FAPbI_3$ obtained from ICSD database (card number 250736, blue)

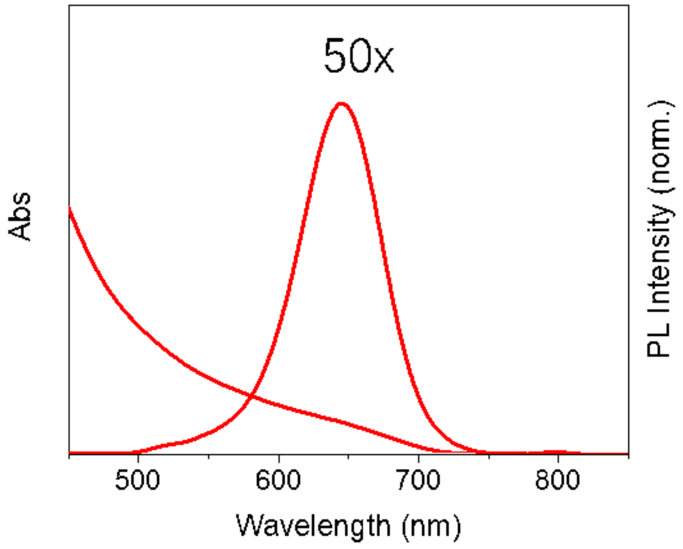

Figure S5: Absorption and PL spectra of $FAPbI_3$ nanoplatelets obtained by scale-up (50 times) synthesis.

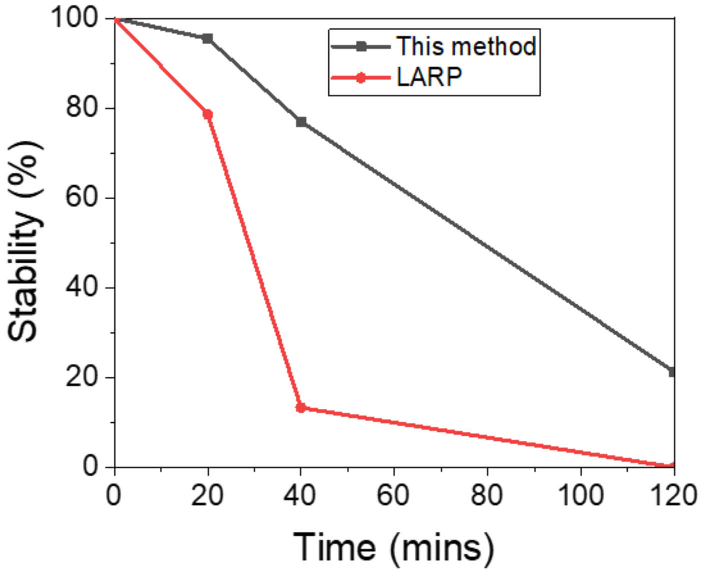

Figure S6**:** Comparision of the stability of NCs prepared by the spontaneous crystallization (current method) and conventional LARP under UV illumination (365 nm, 12W) at ambient conditions: Change in the relative PL intensity of perovskite NC solutions vs UV illumination time.

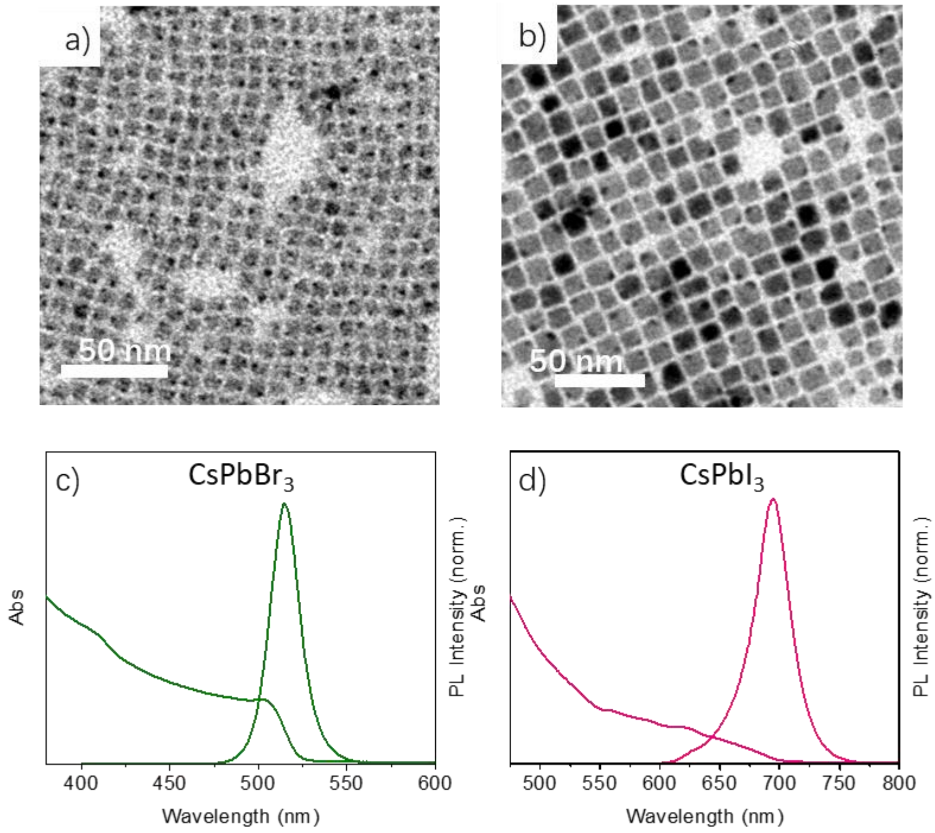

Figure S7**:** TEM images of $CsPbBr_3$ (a) and $CsPbI_3$ (b) nanocubes with corresponding absorption and PL spectra depicted in (c) and (d), respectively. Excitation at 450 nm and 550 nm, respectively.

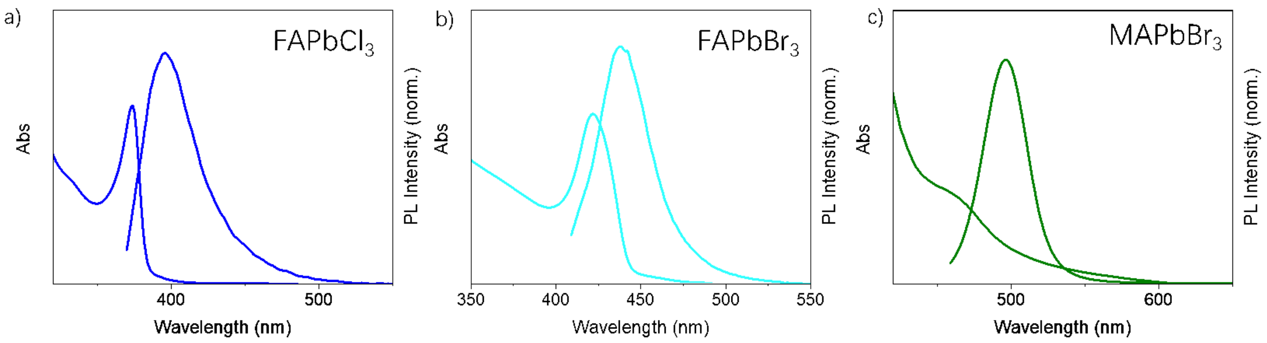

Figure S8: Absorption and PL spectra of $FAPbCl_3$ (a), $FAPbBr_3$ (b), and $MAPbBr_3$ (b) NCs prepared by spontaneous crystallization. Excitation at 350 nm, 400 nm, and 450 nm, respectively.
